# Toll-like receptor expression in severe asthma with chronic rhinosinusitis

**DOI:** 10.1186/2045-7022-3-S1-O2

**Published:** 2013-05-03

**Authors:** Carina Malmhäll, Apostolos Bossios, Margareta Sjöstrand, Bo Lundbäck, Jan Lötvall, Madeleine Rådinger

**Affiliations:** 1 University of Gothenburg, Sahlgrenska Academy, Institute of Medicine, Krefting Research Centre, Sweden

## Background

We have in an epidemiological study identified a group of individuals with multiple asthma symptoms (MSA) that reflect a more severe and uncontrolled disease. An altered innate immunity and adaptive immunity, and effects of microorganisms may play a role in the development of both CRS and asthma. The innate immune system use Toll-like receptors (TLRs) to recognize microbes and activate defense mechanisms within minutes of microbial invasion which is followed by an antigen-specific response by the adaptive immune system. Thus, innate and adaptive immune cells are sequentially activated and reciprocally regulate one another. Glycogen synthase kinase-3β (GSK3β) has been demonstrated as a regulator of both innate and adaptive immunity. GSK3β was shown to modulate inflammatory responses through TLR-mediated production of pro-inflammatory cytokines and inactivation of GSK3β (phosphorylated GSK3β) lead to a reduced IL-12 production, which was suggested to skew the balance towards a Th2 response.

## Aim

To determine the degree of expression of TLRs 2, 4, 7 and 9 on monocytes as well phGSK3β using flow cytometry. We hypothesize that the co-existence of CRS in patients with asthma and especially patients with MSA has an increased TLR expression involved in the innate immune system.

## Method

Participants were selected from an epidemiological cohort, the West Sweden Asthma Study. Clinical parameters as well as fresh peripheral blood cells were obtained from one group of non-asthmatic subjects with CRS (CRS) and four different groups of asthmatics: 7 subjects with MSA (MSA), 7 subjects with other asthma (OA), 7 subjects with MSA and CRS (MSA/CRS) and 9 subjects with OA and CRS (OA/CRS). These five groups were compared to a control group consisting of 10 healthy subjects without asthma or CRS.

## Results

Individuals with MSA or CRS only, showed significantly increased TLR2, TLR7 and TLR9 expression (rMFI) on CD14^+^ monocytes compared to controls as well as the expression of phGSK3β and CD14. Individuals in the combined MSA/CRS group showed increased expression of CD14 and TLR2, but increased expression of TLR4 was only found in the CRS group.

## Conclusion

The upregulated expression of TLRs in the MSA group compared to control group suggest a higher susceptibility towards an altered immune response that might reflect the degree of severity. Furthermore, the increased phosphorylation of GSK3β may indicate a switch in the immune response from a pro-inflammatory to a dysregulated Th2 response.

